# Bacterial Surface Appendages Modulate the Antimicrobial Activity Induced by Nanoflake Surfaces on Titanium

**DOI:** 10.1002/smll.202310149

**Published:** 2024-01-17

**Authors:** Xiayi Liu, Mohd I. Ishak, Huan Ma, Bo Su, Angela H. Nobbs

**Affiliations:** Bristol Dental School Research Laboratories, University of Bristol, Dorothy Hodgkin Building, Whitson Street, Bristol, BS1 3NY, UK; School of Chemistry, Centre for Organized Matter Chemistry and Centre for Protolife Research, University of Bristol, Bristol, BS8 1TS, UK; Bristol Dental School Research Laboratories, University of Bristol, Dorothy Hodgkin Building, Whitson Street, Bristol, BS1 3NY, UK

**Keywords:** bacteria, flagella, implant, nanotopography, titanium, type-1 fimbriae

## Abstract

Bioinspired nanotopography is a promising approach to generate antimicrobial surfaces to combat implant-associated infection. Despite efforts to develop bactericidal 1D structures, the antibacterial capacity of 2D structures and their mechanism of action remains uncertain. Here, hydrothermal synthesis is utilized to generate two 2D nanoflake surfaces on titanium (Ti) substrates and investigate the physiological effects of nanoflakes on bacteria. The nanoflakes impair the attachment and growth of *Escherichia coli* and trigger the accumulation of intracellular reactive oxygen species (ROS), potentially contributing to the killing of adherent bacteria. *E. coli* surface appendages type-1 fimbriae and flagella are not implicated in the nanoflake-mediated modulation of bacterial attachment but do influence the bactericidal effects of nanoflakes. An *E. coli* Δ*fimA* mutant lacking type-1 fimbriae is more susceptible to the bactericidal effects of nanoflakes than the parent strain, while *E. coli* cells lacking flagella (Δ*fliC*) are more resistant. The results suggest that type-1 fimbriae confer a cushioning effect that protects bacteria upon initial contact with the nanoflake surface, while flagella-mediated motility can lead to elevated membrane abrasion. This finding offers a better understanding of the antibacterial properties of nanoflake structures that can be applied to the design of antimicrobial surfaces for future medical applications.

## 1 Introduction

The demand for medical implants is expected to rise due to the increase in average life expectancy and changing lifestyles over the coming decades.^[1]^ Due to the complexity and high cost of implant surgery, most implants are designed to function in the human body for over 10 years. Consequently, successful implantation with good tissue integration that remains infection free is of great importance. Over the past few decades, general improvements in surgical hygiene and implant design have increased implant performance and longevity. However, implant failure remains prevalent.^[2]^ One of the reasons for this failure is due to bacterial infection, resulting in a rising number of revision surgeries.^[3]^

Bacterial attachment and biofilm development are the essential steps in implant-associated infections. Following attachment to the implant surface, bacteria multiply and become embedded within a matrix of extracellular polymeric substances as the biofilm matures. This serves to promote bacterial retention and confers protection against disinfectants and antibiotics.^[4]^ Thus, inhibiting biofilm formation is one of the strategies to combat implant-associated infections. In recent years, technological advancements have enabled antimicrobial agents to be incorporated into implant materials, including metal ions,^[5–7]^ antibiotics,^[8]^ and drug-releasing polymers.^[9]^ However, such chemical approaches have several inherent drawbacks including uncontrolled antibiotic release or depletion, which can serve to promote the emergence of antimicrobial resistance, cytotoxicity and environmental pollution.^[10]^

The idea of using nanotopography to prevent bacterial infection is inspired by nature. For example, naturally occurring antibacterial surfaces like cicada wings, shark skin and gecko feet have been shown to prevent contamination from biofilm formation.^[11]^ Moreover, several studies have found that biomimetic surfaces with similar nanotopographies can halt bacterial attachment or kill the adherent bacteria directly, offering the potential to be exploited as anti-infective materials.^[11]^ Several 1D nanosized features such as pillars,^[12,13,14]^ wires,^[15]^ spikes,^[16]^ and rods^[17]^ with antibacterial properties have been considered, but comparable studies with 2D nanostructures like flakes, blades, plates or ribbons on titanium (Ti) substrates are limited. 2D nanoflakes are predicted to exhibit bactericidal properties due to their unique nanostructure enabling them to function as a “nano-knife” to damage the bacterial cell membrane^[10]^ and to induce bacterial cell wall deformation. 2D nanoflakes are also expected to have better mechanical stability and robustness than 1D nanostructures.^[10,18]^ Thus, investigation of the antimicrobial properties of 2D nanostructures is warranted, including determining the morphological and physiological responses of adherent bacteria to these surfaces.

To understand the mechanistic basis of antimicrobial nanotopographies, the influence of several substrate surface characteristics has been considered to date, such as surface charge, wettability and topography dimensions.^[19]^ By contrast, the role of bacterial envelope properties in mediating nanostructure interactions has been rarely reported, despite the prominent role of these in facilitating bacterial adhesion.^[20,21]^ Our recent study showed that trypsinized *Escherichia coli* cells were less susceptible to cell membrane disruption on a poly(ethylene terephthalate) nanopillared surface when compared with untreated cells. This implied that *E. coli* surface proteins modulated the bacterium-nanopillar interface and thus the extent of the antimicrobial action.

For *E. coli*, the role of type-1 fimbriae in bacterial attachment and biofilm formation is well established, with adhesion to both biotic and abiotic substrata reported.^[23]^ Type-1 fimbriae are tubular organelles of 7 nm in width and 0.2-2 μm in length that are arranged peritrichously on the bacterial surface.^[24]^ Supporting a role for type-1 fimbriae in nanostructure engagement, Kallas et al. found that *E. coli* mutants defective for the expression of fimbrial subunits FimA and FimH exhibited increased attachment to nanopillared polycarbonate surfaces.^[25]^ Alongside type-1 fimbriae, flagella have also been implicated in affecting nanopillar interactions.^[26]^ Flagella are found in many Gram-negative and -positive bacterial species. A major function of flagella is conferring motility but these appendages have also been associated with critical functions such as biofilm formation,^[27,28]^ adhesion^[29]^ and protein export.^[30]^ A study by Jindai et al. reported that flagella-mediated motility influenced the rate of *E. coli* adhesion to a nanopillar array substrate and thus the rate of nanopillar-mediated killing.^[26]^ These findings suggested that both type-1 fimbriae and flagella should be considered important factors that could significantly affect the dynamics of bacterial interactions with nanostructured substrata and thus the efficacy of any antibacterial effects. To provide a more complete understanding of the antimicrobial mechanisms of nanotopographies, it is thus important to explore the role of type-1 fimbriae and flagella during bacterial survival on nanostructured surfaces.

To better understand and validate bacteria-surface interactions relating to nanoflakes, two different nanoflake surfaces were fabricated on a Ti substrate using a hydrothermal synthesis method. Scanning electron microscopy (SEM), atomic force microscopy (AFM) and transmission electron microscopy (TEM) were utilized to evaluate the surface characteristics of the nanoflake surfaces and the bacterium-nanoflake interface at high resolution. A variety of biochemical assays, including BacTiter-Glo, ROS-Glo H_2_O_2_ assay, SYTO9 and Thioflavin S staining, were used to assess the antifouling and bactericidal capacity of nanoflakes. To elucidate the impact of bacterial properties on bacterial susceptibility, this work was then extended to assess the influence of type-1 fimbriae and flagella on *E. coli*-nanoflake interactions. A wild type (WT) *E. coli* parent strain as well as genetically-modified

## 2 Results

### 2.1 Characterization of Nanoflake Surfaces

The Ti-based nanoflake surfaces were fabricated by alkaline hydrothermal synthesis, with the morphology determined by a dissolution and precipitation/growth process.^[31]^ To avoid morphology fluctuations, constant parameters were maintained with the exception of sodium hydroxide (NaOH) concentration and alkaline etching duration (Table 1). Two nanoflake surfaces were generated, designated NF1 and NF2, and characterized using SEM, TEM, and AFM.

**Figure 1:**
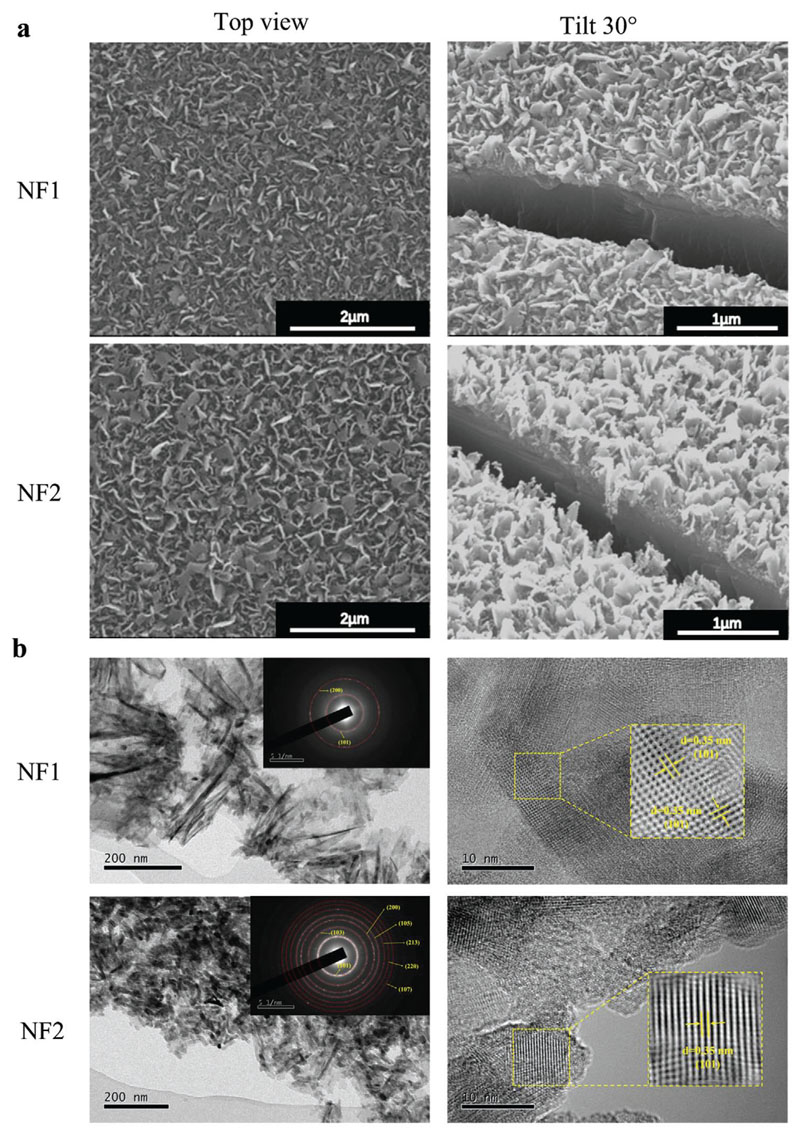
Characterization of nanoflakes on pure Ti substrate. a) Scanning electron micrographs of nanoflake surfaces from a top view and 30° stage tilt. Cracks were deliberately made with the knife to improve the field of view. b) TEM images of NF1 and NF2 with corresponding HRTEM and SAED patterns in insets. Micrographs are representative of three hydrothermal synthesis batches, each batch comprising 22 samples.

Both surfaces exhibited uniform coverage of nanoflakes with different feature sizes (Figure 1a; Figure S1, Supporting Information). Tip thickness was comparable between the two surfaces (24.1 ± 3.2 and 27.4 ± 9.3 nm for NF1 and 2, respectively), but NF2 (457.8 ± 31.1 nm) had taller flakes compared to NF1 (212.5 ± 54.2 nm), which resulted in higher surface roughness and surface area (Table 2; Figure S1, Supporting Information). Also, spacing of the nanoflakes was greater for NF1 (467.3 ± 154.0 nm) than NF2 (187.3 ± 66.7 nm). A wettability analysis of the nanoflake surfaces (Figure S2, Supporting Information) revealed that both were more hydrophilic than the flat control surface, exhibiting a water contact angle *(θ* W) of 16.7° for NF1 and 26.3° for NF2 compared to 46.1° for the control surface. When observed under TEM (Figure 1b), the Selected Area Electron Diffraction (SAED) patterns of both nanoflake surfaces were found to consist of diffraction rings (Figure 1b inset), indicative of a polycrystalline characteristic that can be indexed as TiO_2_ anatase phase. NF2 showed better crystallinity than NF1. High-Resolution Transmission Electron Microscopy (HRTEM) images of the TiO_2_ nanoflakes revealed a lattice fringe with an interplanar spacing of 0.35 nm, which corresponds to the (101) plane of the TiO_2_ phase (Figure 1b inset).

### 2.2 Nanoflake Surfaces Influence Bacterial Viability and Biofilm Formation

**Figure 2:**
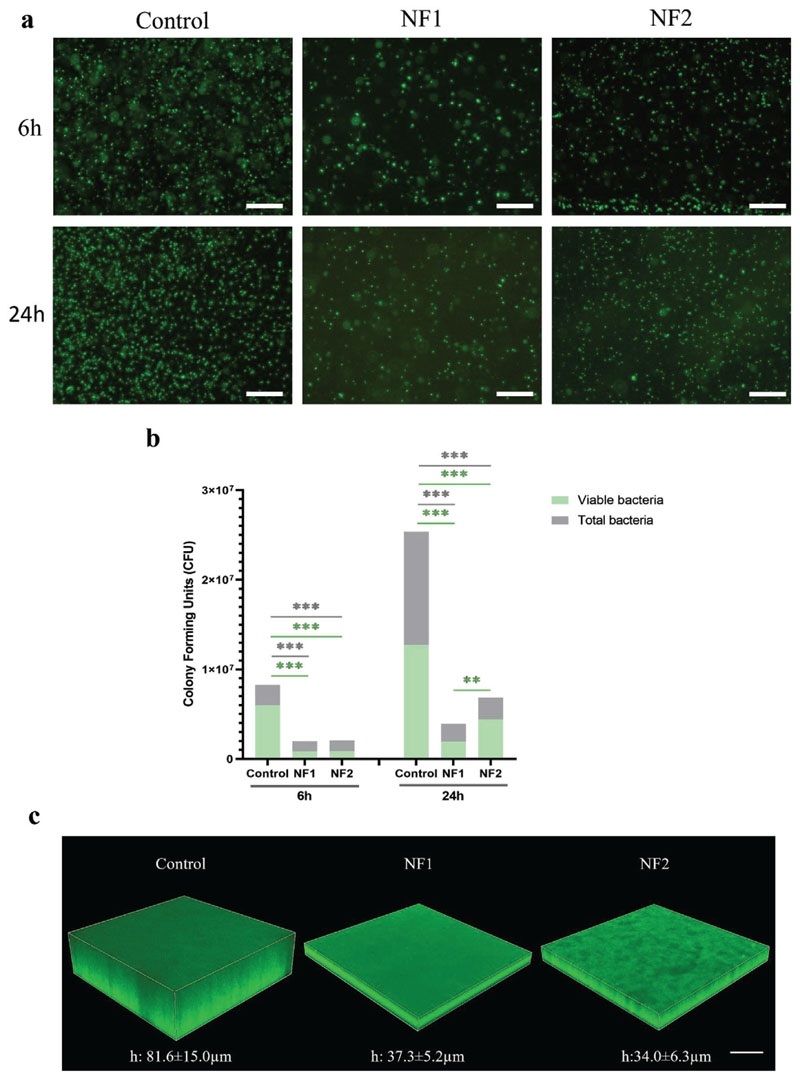
Nanoflake surfaces influence bacterial viability and biofilm formation. a) Representative fluorescence micrographs of *E. coli* on three different surfaces after 6 h (top row) or 24 h (bottom row) incubation. Scale bar, 20 μm. b) Corresponding quantification of bacterial attachment levels based on fluorescence intensity and BacTiter-Glo data. c) Representative confocal laser-scanning microscopy (CLSM) images of 24 h *E. coli* biofilms stained with Thioflavin S on both control and nanoflakes. Scale bar, 60 μm. CFU values are given as mean ± standard deviation. ** *P* < 0.01, *** *P* < 0.001 relative to control, as determined by one-way ANOVA with Tukey HSD post hoc test, n = 3.

The antifouling capacities of nanostructured surfaces have been reported by many groups in recent years.^[32,33]^ To determine if such properties were also conferred by nanoflakes, levels of bacterial attachment to the NF1 and NF2 surfaces were monitored by SYTO9 staining, enabling both visualization and quantification of bacterial biomass. Levels of *E. coli* attachment to NF1 and NF2 were reduced by approximately 79% and 75%, respectively, relative to flat control after 6 h incubation (Figure 2a,b; Figure S3a, Supporting Information). This reduction in bacterial biomass was supported by SEM images for *E. coli* after 6 h (Figure S4). A similar pattern of bacterial attachment was seen after 24 h, providing evidence that both nanoflake surfaces had antifouling and bacteriostatic effects against *E. coli* (Figure 2a,b; Figure S3a, Supporting Information).

Alongside the antifouling and bacteriostatic effects, the potential bactericidal capabilities of the nanoflake surfaces were explored, using the BacTiter-Glo assay to assess bacterial viability. Following 6 h incubation, 72% of *E. coli* cells bound to the control surface were viable, compared to only 42% and 41% for NF1 and NF2, respectively. This implied that after 6 h, the nanoflake surfaces induced higher levels of bacterial cell death than the control surface. After 24 h, the proportion of viable bacteria on all three surfaces was ≈50–65% (Figure 2b; Figure S3b, Supporting Information).

To better understand the influence of nanoflakes on biofilm formation, *E. coli* biofilms were stained with Thioflavin S to detect amyloid curli fibres, which comprise the major proteinaceous component in biofilms^[34]^ after 24 h of static incubation. The mean thickness of biofilms formed on NF1 and NF2 surfaces was reduced by 54% and 58%, respectively, compared to the control (Figure 2c). Fluorescence intensity readings further supported a significant reduction in biofilm biomass on both NF1 and NF2 surfaces relative to the control surface (Figure S5, Supporting Information).

### 2.3 Nanoflakes Induce Bacterial Envelope Rupture and Oxidative Stress Response

**Figure 3:**
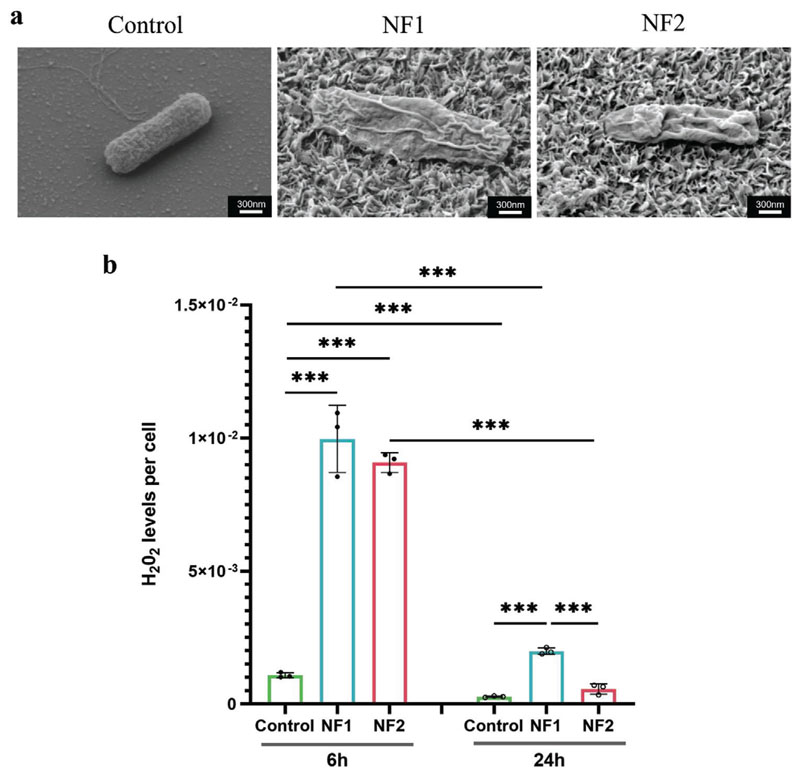
Nanoflake surfaces induce bacterial envelope rupture and oxidative stress response. a) Representative scanning electron micrographs of *E. coli* following 6 h incubation on control, NF1 or NF2 surfaces. Scale bar, 300 nm. b) Levels of H_2_O_2_ within the bound bacterial population, as determined by ROS-Glo assay following 24 h incubation on control, NF1 and NF2 surfaces. Data are presented as mean ± standard deviation. *** *P* < 0.001, as determined by one-way ANOVA with Tukey HSD post hoc test, n = 3.

High-resolution SEM was performed to determine whether the reduction in *E. coli* viability on nanoflake surfaces was caused by a direct bacterium-nanoflake physical interaction. *E. coli* cells on the flat control surface remained intact and potential anchoring by surface appendages such as flagella was observed (Figure 3a). Intact cells were also seen on the nanoflake surfaces but additionally there was evidence of bacterial envelope rupture on both NF1 and NF2 surfaces (Figure 3a).

To investigate if nanoflakes could mediate bactericidal effects by inducing oxidative stress, as had been reported for nanopillars,^[35]^ H_2_O_2_ levels within bacterial cells after 6 h and 24 h incubation on the nanoflake and flat control surfaces were compared via ROS-Glo H_2_O_2_ Assay. H_2_O_2_ levels per cell were 9.3- and 8.4-fold higher for cells bound to the NF1 and NF2 nanoflake surfaces than on the control surface, respectively, at 6 h (Figure 3b). Overall levels of H_2_O_2_ were diminished by 24 h but remained 7.2-fold higher for cells on NF1 and 2.1-fold higher for cells on NF2 compared to the flat control. These data suggested that reactive oxygen species (ROS)-mediated toxicity is likely a key mechanism by which nanoflakes mediate their bactericidal effects.

### 2.4 Physical Characterization of *E. coli* Fimbriae and Flagella Mutants

Two major surface appendages, type-1 fimbriae and flagella, have been shown to influence the dynamics of *E. coli* attachment and biofilm formation, including on nanostructured surfaces.^[35,36]^ To determine if these bacterial appendages also modulate the susceptibility of *E. coli* to the antimicrobial effects of nanoflakes, Δ*fimA* and Δ*fliC* knockout mutants from the *E. coli* Keio collection were exploited.^[35]^ In addition, *E. coli* Δ*fimA* was complemented with plasmid pDL278-*fimA* to generate strain *E. coli fimA*+, while Δ*fliC* was complemented with pJ211-Hi-WT.^[37]^ Restoration of fimbriae or flagella expression was verified by TEM (Figure S6, Supporting Information), but it was noted that the *fimA*+ strain expressed type-1 fimbriae that were seemingly shorter than those seen for the parent strain.

**Figure 4:**
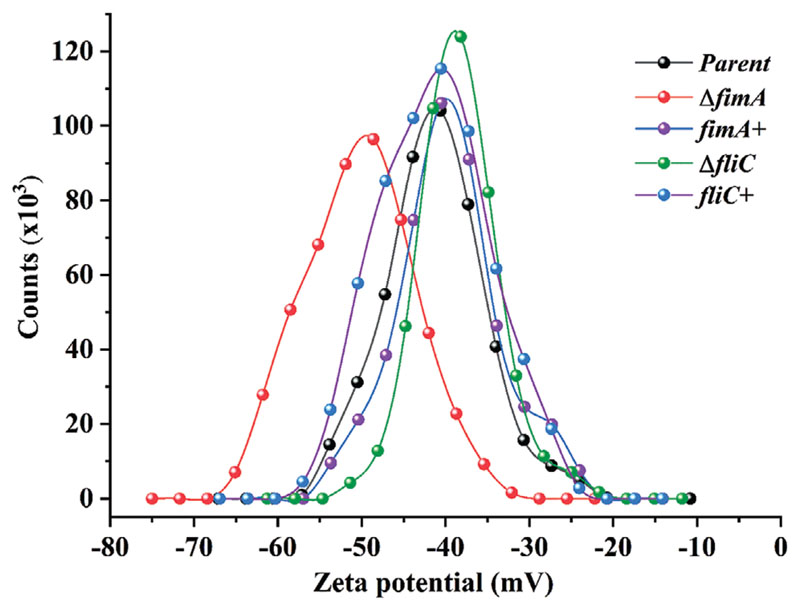
Physical characterization of *E. coli* type-1 fimbriae and flagella mutants. Surface charge profiles for parent, fimbriae and flagella mutant strains.

Since alteration of bacterial surface structures can affect the physicochemical properties of the cell,^[22]^ which in turn could affect bacteria-surface interactions, the impact of mutagenesis on bacterial surface charge was determined (Figure 4). As measured with Dynamic Light Scattering, deletion of type-1 fimbriae increased the negative charge of the cells relative to parent, whilst the complemented *fimA*+ strain and both flagella mutants had comparable profiles to the parent (Figure 4, Table 3). This indicated that loss of fimbriae but not flagella can slightly change the bacterial cell surface charge.

### 2.5 Effects of Type-1 Fimbriae on Bacteria-Nanoflake Interactions

**Figure 5:**
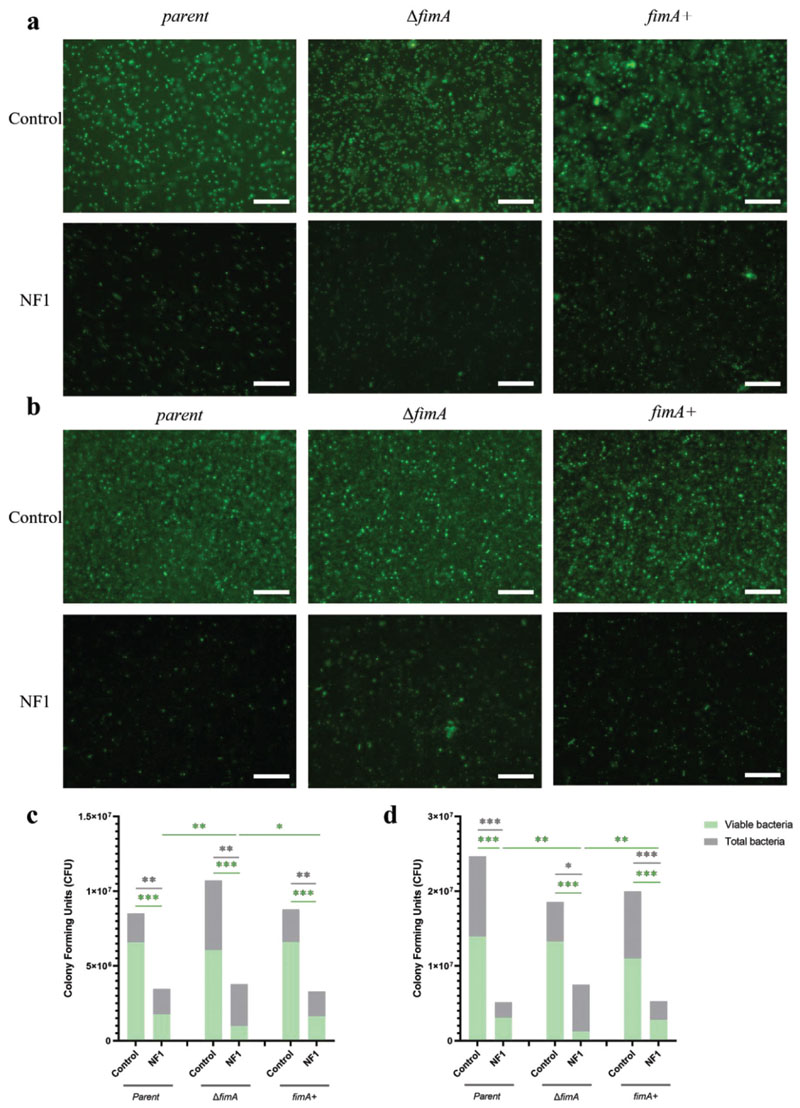
Effects of type-1 fimbriae on bacteria-nanoflake interactions. Representative fluorescence micrographs of *E. coli* parent, Δ*fimA* and *fimA*+ strains on control (top row) and NF1 (bottom row) surfaces after a) 6 h and b) 24 h incubation; scale bar, 20 μm. Numbers of total and viable CFU, as determined by SYTO9 staining and BacTiter-Glo assay, following incubation for (c) 6 h or (d) 24 h. Values are given as mean ± standard deviation. * *P* < 0.05, ** *P* < 0.01 or *** *P* < 0.001, as determined by one-way ANOVA with Tukey HSD post hoc test, n = 3.

**Figure 6:**
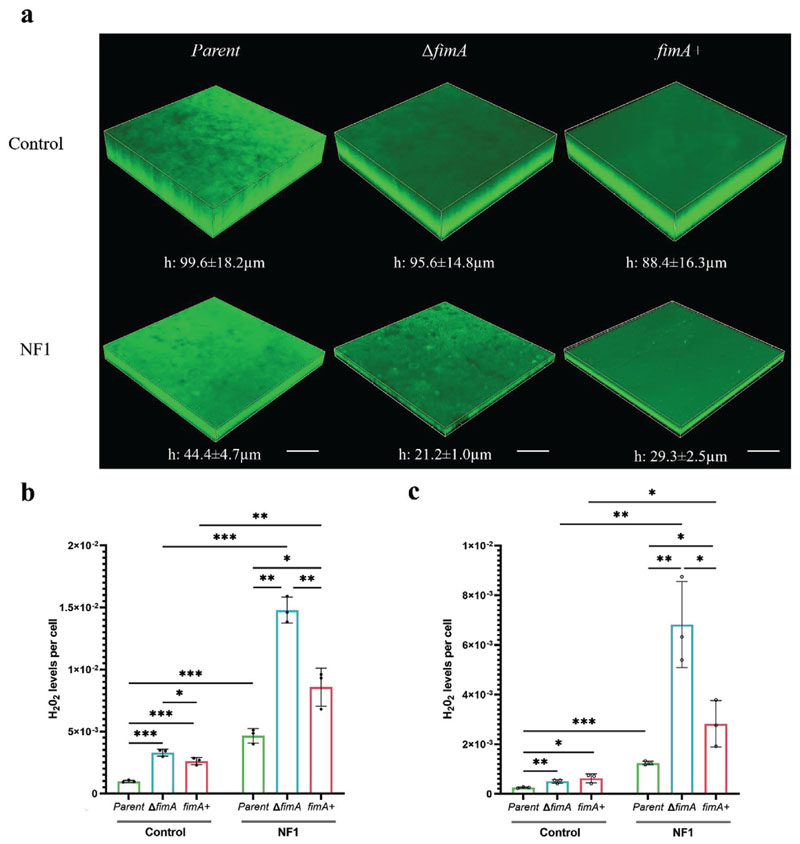
Effects of type-1 fimbriae on the bactericidal activity of nanoflake surfaces. (a) Representative CLSM micrographs of *E. coli* parent, Δ*fimA* and *fimA*+ biofilms formed on control (top row) and NF1 (bottom row) surfaces after 24 h and stained with Thioflavin S; scale bar, 60 μm. Levels of H_2_O_2_ within the bound bacterial population, as determined by ROS-Glo assay, following incubation for (b) 6 h or (c) 24 h. Values are given as mean ± standard deviation. *P < 0.05, **P < 0.01 or ***P < 0.001, as determined by one-way ANOVA with Tukey HSD post hoc test, n = 3.

The potential impact of bacterial surface appendages on the susceptibility of *E. coli* to nanoflakes was first investigated by incubating the type-1 fimbriae mutants on the NF1 surface for 6 h and 24 h. The resulting biomass was then quantified utilizing SYTO9 staining. As seen previously, the presence of nanoflakes reduced overall levels of bacterial attachment by ≈60–65% relative to the control surface at 6 h (Figure 5a,c; Figure S7a, Supporting Information), and by 60–80% at 24 h (Figure 5b,d; Figure S7c, Supporting Information). Loss of type-1 fimbriae had no significant effect on bacterial adhesion to either surface. By contrast, after 6 h, the Δ*fimA* strain exhibited significantly lower levels of viability on the NF1 surface compared to the parent strain (Figure 5c; Figure S7b, Supporting Information). The viability of the *fimA*+ strain was higher than for Δ*fimA* but not restored fully to parent strain levels. A similar trend was seen after 24 h incubation (Figure 5d; Figure S7d, Supporting Information), where viability of the Δ*fimA* mutant was only 26% on the NF1 surface compared to 51% for the parent strain. Moreover, the impact of the Δ*fimA* mutation was specific to nanoflakes, as viability levels were comparable across all three strains on the control surface. To gain additional insight into how type-1 fimbriae affected the bacterial response to nanoflakes, biofilm formation was determined for each strain. Biofilm development was impaired on NF1 after 24 h for all three bacterial strains, with the Δ*fimA* strain forming the thinnest biofilm (Figure 6a). Fluorescence intensity readings supported these observations, although statistical significance was not reached (Figure S8, Supporting Information).

To determine whether the heightened sensitivity of the Δ*fimA* mutant to nanoflakes was associated with the oxidative stress response, H_2_O_2_ levels across the three stains were measured. At both 6 h and 24 h, all three strains had elevated levels of H_2_O_2_ when bound to the NF1 surface compared to the control, but overall levels were significantly higher for NF1 within the Δ*fimA* cells than in the parent or *fimA*+ cells (Figure 6b,c). Taken together, these data imply that type-1 fimbriae play an important role in modulating the interactions and thus antibacterial effects of nanoflakes on *E. coli*, including those mediated by ROS.

### 2.6 Effects of Flagella on Bacteria-Nanoflake Interactions

**Figure 7:**
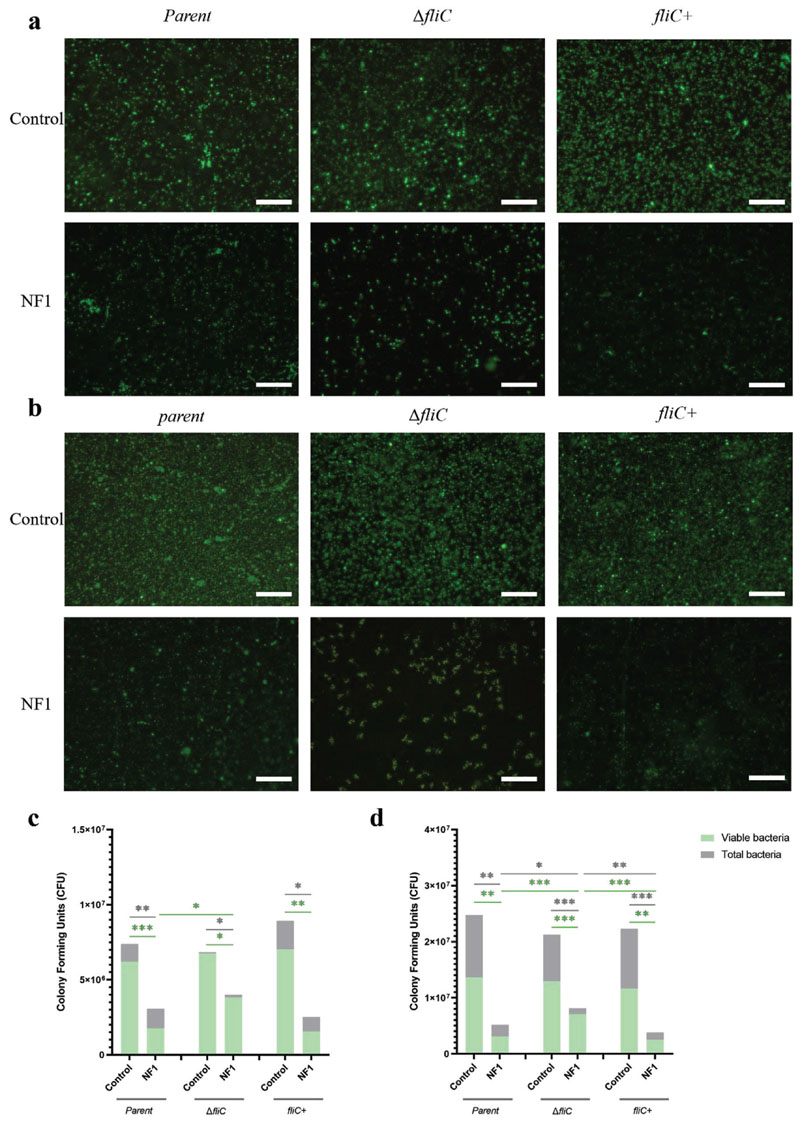
Effects of flagella on bacteria-nanoflake interactions. Representative fluorescence micrographs of *E. coli* parent, Δ*fliC* and *fliC*+ strains on control (top row) and NF1 (bottom row) surfaces after a) 6 h and b) 24 h incubation; scale bar, 20 μm. Numbers of total and viable CFU, as determined by SYTO9 staining and BacTiter-Glo assay, following incubation for (c) 6 h or (d) 24 h. Values are given as mean ± standard deviation. *P < 0.05, ** *P*< 0.01 or ***P < 0.001, as determined by one-way ANOVA with Tukey HSD post hoc test, n = 3.

**Figure 8:**
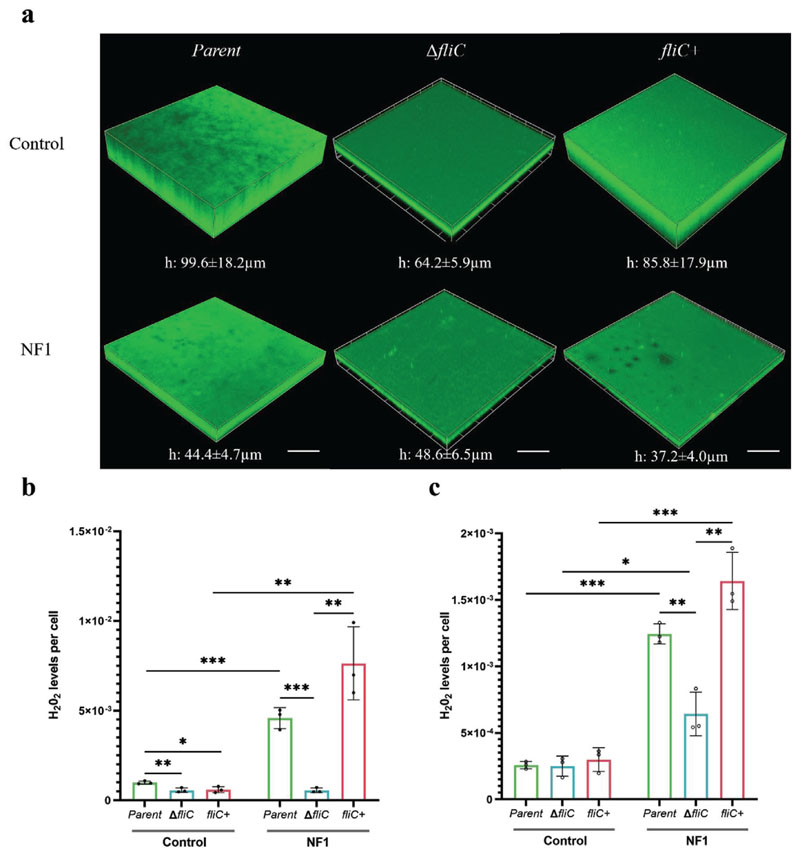
Effects of flagella on the bactericidal activity of nanoflake surfaces. a) Representative CLSM micrographs of *E. coli* parent, Δ*fliC* and *fliC*+ biofilms formed on control (top row) and NF1 (bottom row) surfaces after 24 h and stained with Thioflavin S; scale bar, 60 μm. Levels of H_2_O_2_ within the bound bacterial population, as determined by ROS-Glo assay, following incubation for b) 6 h or c) 24 h. Values are given as mean ± standard deviation. *P < 0.05, **P < 0.01 or ***P < 0.001, as determined by one-way ANOVA with Tukey HSD post hoc test, n = 3.

Similar to type-1 fimbriae, loss of flagella did not significantly affect bacterial adhesion to either surface, as attachment levels were comparable for the parent, Δ*fliC* and *fliC*+ strains, although the antifouling effects of the nanoflakes were retained at both 6 h and 24 h (Figure 7a–d; Figure S9a,c, Supporting Information). However, in direct contrast to the effects seen for type-1 fimbriae, at 6 h, for cells lacking flagella, the proportion of viable cells was comparable between the NF1 and control surfaces, whereas the presence of nanoflakes significantly reduced the viability of both the parent and complemented strains by ≈75% (Figure 7c; Figure S9b, Supporting Information). A similar trend was also seen at 24 h (Figure 7d; Figure S9d, Supporting Information). Again, levels of viability across the three strains were comparable on the control surface. However, the mean thickness of the Δ*fliC* biofilm on the control surface was significantly reduced relative to the parent and *fliC*+ strains, which correlates with the known role of flagella in promoting biofilm formation (Figure 8a; Figure S10, Supporting Information). This difference was not seen between the biofilms formed on NF1, likely because the effects of losing structural support from flagella was counteracted by differences in levels of intracellular ROS (Figure 8a; Figure S10, Supporting Information). The Δ*fliC* cells had the lowest levels of H_2_O_2_ of the three bacterial strains when bound to the NF1 surface, while levels in the parent and *fliC*+ strains were comparable (Figure 8b,c). As for type-1 fimbriae, these data indicated that the presence of flagella influence the capacity for nanoflakes to induce oxidative stress.

## 3 Discussion

### 3.1 Antibacterial Mechanisms of 2D Nanoflake Surfaces

Investigating the mechanisms that govern bacterial survival on nanostructured Ti substrata is of vital importance for the development of antimicrobial nanotopographies that can be applied to medical implants. To date, synthetic nanostructured surfaces inspired by nature have been shown to kill adherent bacteria, especially Gram-negative species.^[22,38–40]^ 2D nanostructures like nanoplatelets and nanoflakes can exhibit nanoknife functions,^[18,41,42]^ and asymmetrical nanosheets on Ti sub-strate have been shown to mediate bacterial killing,^[43]^ indicating the potential of 2D nanostructures as antibacterial surfaces. Although reduced bacterial viability and membrane rupture on 2D nanostructures have been reported previously,^[40,44]^ full understanding of the mechanisms that underpin the antibacterial properties of the nanostructures and design considerations for optimal performance are still needed. Thus, we evaluated the bacterial response to two nanoflake surfaces generated by hydrothermal synthesis to assess their antimicrobial potential and mechanism(s) of action.

The final shape and dimensions of the nanostructures were dependent on a variety of synthesis parameters during alkaline hydrothermal synthesis, particularly alkaline concentration and etching duration.^[31,45]^ To obtain nanoflakes, a lower NaOH concentration (0.5 M and 0.75 M) was selected, which enabled more oxide growth than at higher concentrations.^[46]^ Higher flake density on NF2 was also achieved by using a more concentrated NaOH solution during the etching process compared with NF1.^[47]^ In this study, NaOH etching duration was found to decrease flake-edge length, which is consistent with previous work.^[43]^ According to the mechanobactericidal model,^[48]^ the sharper the flake edge, the greater the extent of bacterial cell membrane stretching and possible rupture. The data reported here suggest that the nanoflake edge in this study was sharp enough to rupture and kill a small proportion of the adherent bacteria, with other factors such as strength of attachment, bacterial cell dimensions, surface structure and orientation at the point of contact contributing to the outcome.

Both nanoflake surfaces showed significant antifouling and bacteriostatic effects against *E. coli* over the 24-h period, with significantly lower numbers of bacteria bound to the nanoflake surfaces than to the flat control. These properties likely derive from a combination of the intermediate surface roughness and nanoflake density of the substrata. Previous reports on other protruding nanotopographies have shown that intermediate surface roughness (30 nm-1 μm) can reduce bacterial attachment by minimizing the number of surface contact points.^[10,49]^ Furthermore, the nanoflake density on the surfaces was such that it prevented individual bacterial cells from attaching inbetween the nanoflakes, which would also be expected to contribute to the antifouling behaviour.^[50,51]^

There was also evidence of the nanoflakes mediating bactericidal effects, as even when accounting for differences in numbers of adherent cells across the flat and nanoflake surfaces, levels of bacterial viability were significantly reduced on both nanoflake surfaces relative to the flat control. The thickness of both nanoflakes was between 15–35 nm, with most in a perpendicular orientation. Similar to the nanospikes, this “sharp” wedge of nanoflakes could be sufficient to induce mechanobactericidal behaviour.^[52]^ Nanoflake density could affect bactericidal activity, as the spacing between flake tips can lead to different levels of bacterial cell envelope stretching between contact points.^[10,44]^ This can potentially explain why NF1, which had a greater interflake distance, showed better bactericidal activity against *E. coli* compared to NF2. However, there was only limited evidence of cell rupture in our study and rather, the data implied that the principal mechanobactericidal mechanism may be due to membrane deformation and subsequent induction of an oxidative stress response. Bacterial contact with carbon nanotubes (CNTs) or Ti nanopillars has been shown to promote the accumulation of intracellular ROS, and this correlated with evidence of cell membrane deformation.^[53,54]^ Levels of endogenous ROS were significantly higher for bacteria attached to the nanoflake surfaces compared to the flat surface. Since the induction of oxidative stress can potentially cause considerable damage to cellular components and ultimately result in bacterial cell death,^[55–57]^ this mechanism could underpin the bactericidal effects of the nanoflakes seen following initial contact.

The scale of the ROS response did diminish overall from 6 h to 24 h. This may indicate that for bacteria that attach later to areas that have already been colonized, the impact of the nanoflakes and thus oxidative stress response is diminished. Nonetheless, H_2_O_2_ levels remained significantly higher for bacteria bound to the nanoflakes than to the flat surface at 24 h, particularly for the NF1 surface. Since endogenous ROS is reported to be self-amplifying^[58]^ and can also be secreted by bacteria into the surrounding environment as a signalling molecule,^[59,60]^ this may indicate that bacteria that had direct contact with nanoflakes can sustain and amplify ROS production, even after initial attachment. Those bacteria can then produce and secrete more ROS into the surrounding environment, which in turn can affect even those bacteria that are not in direct contact with the nanostructures. At 24 h, comparable proportions of viable cells were recorded across the control and nanoflake surfaces but differences were seen in total and adherent cell numbers over the 24 h period. This suggested that, in the later stages, elevated ROS levels on the nanoflake surfaces may have restricted bacterial replication rather than directly leading to bacterial cell death.^[61,62]^

In summary, both NF1 and NF2 surfaces exhibited antifouling and bactericidal effects against *E. coli* within the first 6 h, with the latter effect correlating with elevated levels of oxidative stress. After 24 h, the bactericidal effects had diminished and rather, in these later stages, nanoflake surfaces exhibited both antifouling and bacteriostatic effects that, in turn, hampered bacterial biofilm formation.

### 3.2 The Influence of Bacterial Surface Appendages During Bacteria-Nanoflake Interactions

We recently demonstrated that the bactericidal activity of nanostructured surfaces may be influenced by bacterial surface proteins during the initial stages of attachment.^[22]^ To further understand this complex interaction between bacteria and nanostructures, here we explored the importance of type-1 fimbriae and flagella, two surface determinants of *E. coli* that had already been associated with influencing attachment to nanostructures, in modulating the outcome of interactions with nanoflakes. Neither loss of type-1 fimbriae nor flagella from the cell surface affected overall *E. coli* attachment levels,^[63]^ potentially indicating that redundancy of function across the surface appendages meant that others were able to compensate. Another explanation is that the contribution of both appendages on bacterial attachment is surface-type specific, given that this study is the first to investigate their role in binding to 2D nanoflakes. However, cells lacking type-1 fimbriae exhibited higher levels of cell death when specifically bound to the nanoflakes, while the inverse was seen for cells lacking flagella. This implied that type-1 fimbriae may confer protection against nanoflake-mediated killing, whilst the presence of flagella can exacerbate this killing.

For type-1 fimbriae, one possible explanation is that the fimbriae may function as an “air-bag” that, upon initial surface contact, restricts the extent of direct contact between the bacterial cell envelope and the nanoflakes. A similar protective effect has been proposed for bacteria using fimbriae to evade contact with antibiotics during initial infection.^[64]^ By minimizing the extent of bacterial membrane deformation by nanoflakes, this would, in turn, impair the induction of oxidative stress. By contrast, for flagella, the effects likely relate to the motility conferred by flagella, as proposed by Jindai et al.^[26]^ It is predicted that motility following bacterial attachment to nanoflakes will exacerbate physical disruption of the bacterial cell envelope and thus accelerate nanoflake-mediated killing.

**Figure 9:**
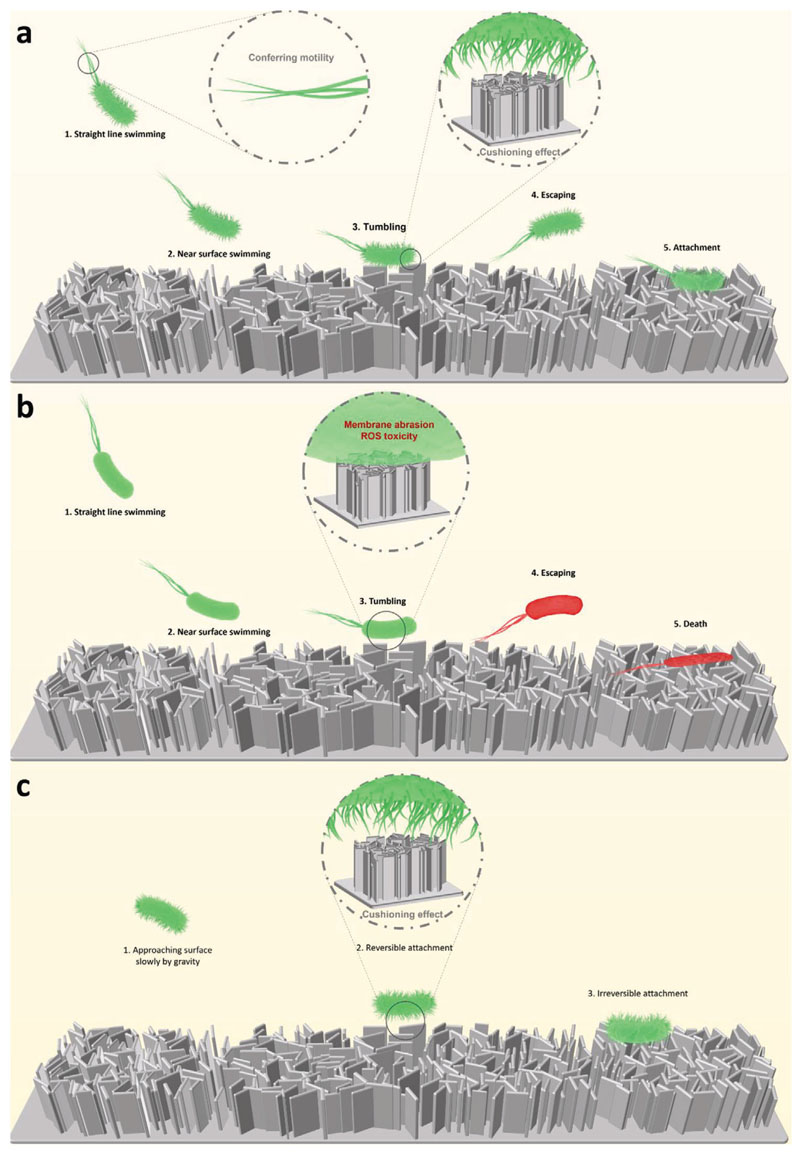
Proposed mechanisms underpinning bacteria-nanoflake surface interactions. a) Attachment of wild-type bacteria to the nanoflake surface. b) Influence of fimbriae loss on bacterial-membrane interaction. c) Influence of flagella loss on bacterial-membrane interaction.

In summary, the data presented here support the following model to describe bacteria-nanoflake interactions (Figure 9). Bacteria such as *E. coli* under planktonic conditions will swim in a straight line by rotating the flagella motor behind the cell body.^[65]^ After identifying and approaching the surface, part of the flagella motor is switched, allowing the bacterium to tumble and find the optimal point on the surface to anchor. During this near-surface swimming phase, surface torque is increased because of the drag on the cell membrane near to the nanoflakes.^[66]^ In this case, the presence of type-1 fimbriae adds an extra repulsive force between the bacterial membrane and surface, physically holding the bacterial cell away from the nanoflakes and reducing direct contact with the nanoflakes.^[63]^ However, this initial fimbriae-mediated protective effect can ultimately be overcome by the adhesive properties of flagella and other bacterial surface adhesins. Motility conferred by flagella can then further exacerbate the antibacterial effects of the nanoflakes by contributing to shear forces at the bacterial cell-nanoflake interface, enhancing cell membrane deformation, potential lysis and induction of oxidative stress. During this state, which is maintained until firm attachment is initiated, flagella and type-1 fimbriae affect the bacteria-surface interaction inversely.

## 4 Conclusion

This study found that nanoflake surfaces mediated significant antifouling and bactericidal effects against *E. coli* up to 24 h, highlighting the potential for such 2D nanostructured surfaces to serve as promising candidates for anti-infective implant materials. Surface roughness, nanoflake density and height were parameters that contributed to the antibacterial properties of the nanoflake surfaces, with induction of ROS-mediated toxicity a major driver of the bactericidal activity. This study also demonstrated the importance of two bacterial appendages, type-1 fimbriae and flagella, in modulating the bacterial response to nanoflake surfaces. It is proposed that type-1 fimbriae can protect bacteria from the nanoflake surface via a cushioning effect, while flagella-mediated motility has the opposing effect of increasing *E. coli* susceptibility to the nanostructured surface. Such factors should be considered when optimizing the design of nanostructured surfaces for biomedical applications to improve their antimicrobial efficacy.

## 5 Experimental Section

### Generation of Nanoflakes on Ti Substrate by Hydrothermal Synthesis

Grade 5 pure Ti discs were mechanically polished (Struers TegraForce-1) on MD-Largo discs mounted with silicon carbide (SiC) paper (Struers) using decreasing SiC grit sizes (from 50 to 4000). Following mechanical polishing, mirror polishing was achieved using MD Chem pads (Struers) with the addition of 10% hydrogen peroxide (Acros Organics) in colloidal silica suspension (Struers). To remove contaminants, discs were cleaned by ultrasonication in water and ethanol (Merck) for 30 and 15 min, respectively. Then Ti discs were placed on a polytetrafluoroethylene (PTFE) sample holder inside a PTFE vessel (120 cm × 11 cm outer × 9 cm inner) and immersed in different concentrations of NaOH solution. PTFE vessels were put into metal vessels and positioned in a pre-warmed oven at 240 °C for the designated time. Details of the processing settings are given in Table 1. Discs were cooled to room temperature and then washed in water and analytical reagent grade (99.99%) ethanol (Fisher Scientific, MA, USA) for 10 min each. After drying in a fume hood, the discs were placed in a muffle furnace (Elite Thermal Systems Ltd) and heat-treated at 300 °C for 1 h. After cooling, the discs were soaked in a 0.6 M hydrochloric acid (HCl) solution for 1 h for ion exchange. Following the cleaning process, the discs were finally heat-treated at 600 °C for 2 h.

### Nanoflake Surface Characterization using Electron and Atomic Force Microscopy

A FEI Quanta 200 field emission gun scanning electron microscope was used to determine the dimensions of the nanoflakes produced by hydrothermal synthesis. To establish the homogeneity of nanoflake surface coverage and the reproducibility between different hydrothermal synthesis batches, top-view SEM micrographs were taken from five different batches and at three locations per disc. Tilted (40°) SEM micrographs were used to estimate the length and width of the nanoflakes, at three locations per disc, from three different batches. A total of 20 measurements were recorded from each SEM micrograph. These data were averaged from each batch to estimate the mean dimensions of the nanoflakes.

Nanoflakes were scraped off the surface of the Ti using a polycarbonate shard from the Ti substrate and suspended in 2 μL ethanol, before being carefully transferred onto a carbon film-loaded copper grid using a micropipette. The mounted sample was characterized using an FEI Tecnai 20 transmission electron microscope with energy dispersive X-ray spectroscopy. The structure of nanoflakes was analyzed using Digital Micrograph (Gatan) and MDI Jade 6.5 software. Inverse fast Fourier transform was performed to improve the signal-to-noise ratio.

A Multimode III atomic force microscope coupled with a Nanoscope VII controller was utilized to take the AFM images. The samples were examined in tapping mode with ScanAsyst fluid cantilevers from Bruker bearing a silicon nitride tip of 40 nm diameter. The resonance frequency of the cantilever was measured at 150 ± 50 kHz and a spring constant of 0.7 N m^−1^ with back coating of Ti. A total of 20 measurements were recorded from each group, and data was analyzed using Fiji and Gwyddion.

### Bacterial Strains and Culture Conditions

The bacterial strains used for this study are given in Table S1 (Supporting Information). Bacteria were routinely cultured in Luria Bertani broth (BD Biosciences) for 16 h at 37 °C, 220 rpm. As required, broth cultures were supplemented with kanamycin (30 μg mL^−1^) or spectinomycin (50 μg mL^−1^). For use in assays, bacteria were subcultured to OD_600_ 0.1 and grown to mid-exponential phase (≈10^7^ CFU mL^−1^).

### 5.0.0.1. E. coli Mutagenesis

All plasmids and primers used in this study are listed in Table S2 (Supporting Information). Plasmid pDL278-*fimA*^+^ was generated by amplifying a 1068-bp fragment that included the entire *fimA* coding sequence from *E. coli* K12 (BW25113) genomic DNA using primers FimA_compF/compR, which incorporated *Sph*I and *Sac*I restriction sites at the 5’ and 3’ ends of the amplicon, respectively. The amplicon was digested with *Sph*I/*Sac*I and ligated into similarly digested pDL278 using T4 DNA ligase. This construct was purified and used to transform *E. coli* Δ*fimA* to generate strain UB3126. Complementation was confirmed by visualization of type-1 fimbriae using TEM.

To generate the *fliC*+ strain UB3124, the Kan^R^ resistance cassette, *aphA3*, of UB3071 was first removed by transformation with pCP20 and growth of carbenicillin resistant colonies in LB at 30 °C overnight. The temperature-sensitive pCP20 was then removed by growth at 37 °C with-out antibiotics. Successful transformants were identified by susceptibility to kanamycin and carbenicillin and then transformed with plasmid pJ211_WT_hi,^[37]^ with selection on LB agar supplemented with kanamycin (30 μg mL^−1^). Complementation was confirmed by visualization of flagella using TEM.

### Negative Staining

Planktonic cells were grown to mid-exponential phase. Under sessile conditions, bacteria were maintained on LB agar at 37 °C for 24 h before colonies were suspended in LB broth to OD_600_ 1.0 immediately prior to TEM preparation. TEM support grids (Agar Scientific, AGS166) were incubated with a 10 μl drop of bacterial suspension for 3 min and washed in distilled water for 30 s three times. Grids were then fixed in 1% paraformaldehyde-phosphate-buffered saline (PBS) buffer for 5 min. Following a 30 s wash in water, the support grids were incubated in 3% uranyl acetate for 30 s. Grids were then washed in distilled water for 30 s and left to air dry.

### Surface Charge of Bacteria

The zeta potential for different *E. coli* strains was measured using a Malvern Zetasizer Nano-ZS (Malvern Panalytical). *E. coli* cells were adjusted in PBS to OD_600_ 1.0. Cells were then diluted 1:100 before being vortexed and transferred onto a folded capillary zeta cell (DTS1070). The zeta potential was measured using Malvern Zetasizer software (Malvern V7.11) and collected using the “general purpose” mode. Measurements were taken in triplicate from three independent experiments.

### Contact Angle Measurements

Water contact angle measurements on pure Ti nanoflake discs were performed in triplicate using a KRÜSS Drop Shape Analyzer DSA100 instrument with the water drop volume setting at 2.0 μL.

### Determination of Bacterial Viability

BacTiter-Glo (BTG) assay was used to determine the number of metabolically active and thus viable bacteria, as described previously.^[53]^ Briefly, sterile Ti discs were placed into 24-well plates and 30 μl of bacterial suspension (10^5^ CFU) was transferred onto the top of the nanoflake surface. Following incubation at 37 °C under static conditions, each disc was gently washed and transferred to a white 24-well plate. BTG reagent (30 μl) was applied to each disc and after 5 min at room temperature in the dark, the luminescent signal was measured using a Tecan Spark microplate reader. Luminescence was recorded using automatic attenuation, 1 s integration time and 0 s settle time. Quantification of the number of viable bacterial cells on each Ti disc was based on standard curves relating RLU to CFU.^[53]^

### Determination of Bacterial Attachment and Biofilm Formation

Bacteria were incubated on Ti discs, as described above. Following the wash step, 30 μl SYTO 9 (L7007, Thermo Fisher Scientific) was applied to each disc and incubated at room temperature for 15 min in the dark. Excess stain was removed by immersion of each disc in Tris-HCl buffer and discs were then transferred to a black 24-well plate. A Tecan Spark microplate reader was utilized to measure the fluorescence intensity at 20 locations per disc, from which mean fluorescence intensities were calculated. Fluorescence data were then converted to bacterial CFU based on equation: *y* = 6 × 10^−5^*x*^2.4704^ (*R*^2^ = 0.9997). In addition, discs were placed onto glass slides, the upper surface covered with a glass coverslip, and imaged using a Leica DMLB fluorescence microscope, capturing 5 locations per disc using CellSense software (Olympus).

### SEM Sample Preparation and Imaging

Bacteria were incubated on Ti discs, as described above. Following the wash step, adherent bacteria were fixed by immersion of the disc in a 2.5% glutaraldehyde solution dissolved in 0.1 M potassium phosphate buffer overnight at room temperature. Samples were then dehydrated in an ethanol series of 20%, 40%, 60%, 80%, and 100% followed by critical point drying. Discs were mounted onto carbon stubs and sputter coated with 10 nm gold before viewing under SEM.

### CLSM

Custom sample holders were prepared by mounting coverslips onto a poly(methyl methacrylate) (PMMA) slide (length 76 mm; width 26 mm; height 1.5 mm) with three apertures (15 mm diameter). Ti samples were loaded into the apertures and attached to the bottom of the PMMA slide. Then the sealed PMMA slide was inverted and the samples visualized with an SP8-II CLSM (Leica, Germany). Image J and ICY software were used to analyze micrographs.

### ROS-Glo H_2_O_2_ Assay

Induction of oxidative stress was investigated using the ROS-Glo H_2_O_2_ Assay (Promega), which measures levels of intracellular H_2_O_2_. Bacteria were incubated on Ti discs for 18 h, as described above. H_2_O_2_ substrate solution was then applied, according to the manufacturer’s instructions, and the discs were incubated for a further 6 h. ROS-Glo detection reagent was added and incubated at room temperature for 20 min, after which excess solution was removed. Discs were transferred to a white 24-well plate and luminescence measurements were recorded using a Tecan Spark microplate reader. Luminescence was recorded using automatic attenuation, 1 s integration time and 0.15 s settle time.

### Statistical Analysis

Statistical analyses were performed using Graph-Pad Prism (version 9.3.1). For bacterial viability and attachment data, statistically significant differences were determined using a one-way analysis of variance (ANOVA) with a Tukey HSD post hoc test. The threshold for statistical significance was set at *p* < 0.05. Unless otherwise stated, values given are mean ± standard deviation and are representative of three independent experimental replicates (n = 3), performed in triplicate.

## Supplementary Material

Supporting Information is available from the Wiley Online Library or from the author.

Supporting Information

## Figures and Tables

**Table 1:** Settings for hydrothermal synthesis.

	NF1	NF2
NaOH concentration (M)	0.5	0.75
Duration (minutes)	75	60
NaOH volume (ml)	65	65
Temperature (°C)	240	240

*E. coli* strains, with or without type-1 fimbriae or flagella, were evaluated.

**Table 2:** Characterization of nanoflakes and flake size^a)^.

	Roughness (nm)	Surface area (µm)	Height (nm)	Nanoflake spacing (nm)	Tip thickness (nm)
NF1	36.0 ± 0.5	28.9 ± 0.5	212.5 ± 54.2	467.3 ± 154.0	24.1 ± 3.2
NF2	51.4 ± 2.8	54.4 ± 5.7	457.8 ± 31.1	187.3 ± 66.7	27.4 ± 9.3

a) Data were obtained by AFM and analyzed using Gwyddion and Fiji

**Table 3:** Surface charge measurements and relative differences (% change).

	*Parent*	Δ*fimA*	*fimA+*	Δ*fliC*	*fliC+*
Zeta potential (mV)	−40.4±1.9	−46.4±2.8	−37.8±1.3	−38.9±2.0	−41.4±0.5
Percentage change	0	14.9%	−6.4%	−3.6%	2.4%

## Data Availability

The data that support the findings of this study are available from the corresponding author upon reasonable request.
