# Supplementary material for: Bacterial Surface Appendages Modulate the Antimicrobial Activity Induced by Nanoflake Surfaces on Titanium
Source: Small. Author manuscript; Available in PMC 2024 Aug 22. (PMC7616388; doi:10.1002/smll.202310149)
Supplement: Supporting Information [file EMS198109-supplement-Supporting_Information.pdf]

NANO · MICRO small

Supporting Information

for *Small*, DOI 10.1002/smll.202310149

Bacterial Surface Appendages Modulate the Antimicrobial Activity Induced by Nanoflake Surfaces on Titanium

Xiayi Liu, Mohd I. Ishak, Huan Ma, Bo Su and Angela H. Nobbs**

Supporting Information

Bacterial Surface Appendages Modulate the Antimicrobial Activity Induced by Nanoflake Surfaces on Titanium

Xiayi Liu, Mohd I. Ishak, Huan Ma, Bo Su, and Angela H. Nobbs**

Table S1. Bacterial strains used and relevant characteristics

Strain designation	Bacterial strain	Relevant genotype/characteristics	Source/reference
UB2967	*E. coli* K12 (MC4100)	Wild-type	[1]
UB3070	*E. coli* K12 (BW25113)	Parent	[2]
UB3072	*E. coli* K12 (BW25113)	Δ*fimA*; Kan^R	[2]
UB3071	*E. coli* K12 (BW25113)	Δ*fliC*; Kan^R	[2]
UB3126	*E. coli* K12 (BW25113)	Δ*fimA* pDL278-*fimA+*; $Spec^R$	This study
UB3124	*E. coli* K12 (BW25113)	Δ*fliC* pJ211-WT-hi (*fliC+*); Kan^R	This study

Table S2. Plasmids and primers used for bacterial mutagenesis

Primer name	Sequence	
FimA_compF	ACATGCATGCAAAAAGAGAAGAGGTTTGATTTAAC	
FimA_compR	CGCGAGCTCTTATTGATACTGAACCTTGAAGG	
FimA_U	CGTTATACCGCCAGTAATGC	
FimA_R	AACCTGTCCTAACTGAACGG	
**Plasmid name**	**Genotype/Features**	**Source/reference**
pDL278	6.6 kb; Spec^R; ColE1*ori*; *E. coli*–streptococcal shuttle vector	[3]
pDL278-*fimA*$^+$	pDL278 carrying *fimA*	This study
pJ211_WT_hi	*fliC* promoter; pUC origin; Kan^R; expression plasmid for FliC	[4]
pCP20	*FLP*$^+$; λ cI857$^+$; λ p_R Rep^{ts}; Amp^R; Cm^R; temperature-sensitive	[5]

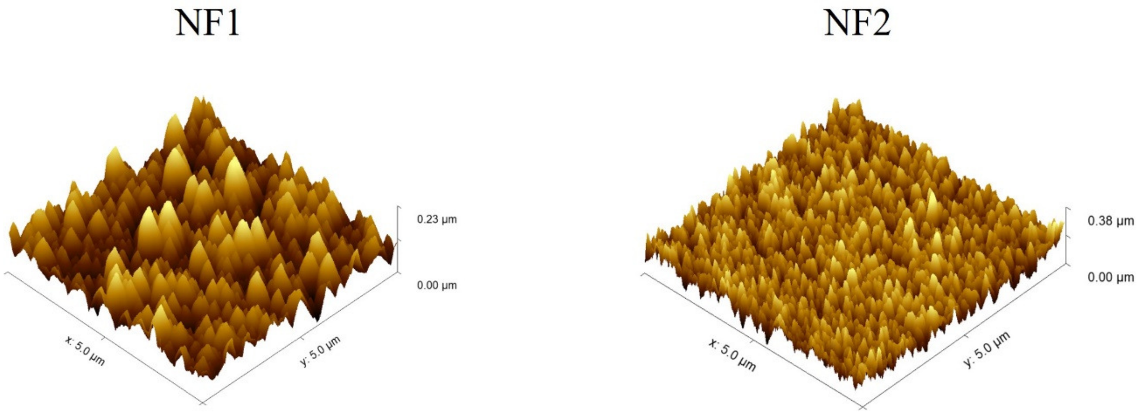

Figure S1. Representative AFM images of NF1 and NF2. Data were obtained by AFM and analyzed by Gwyddion and Fiji.

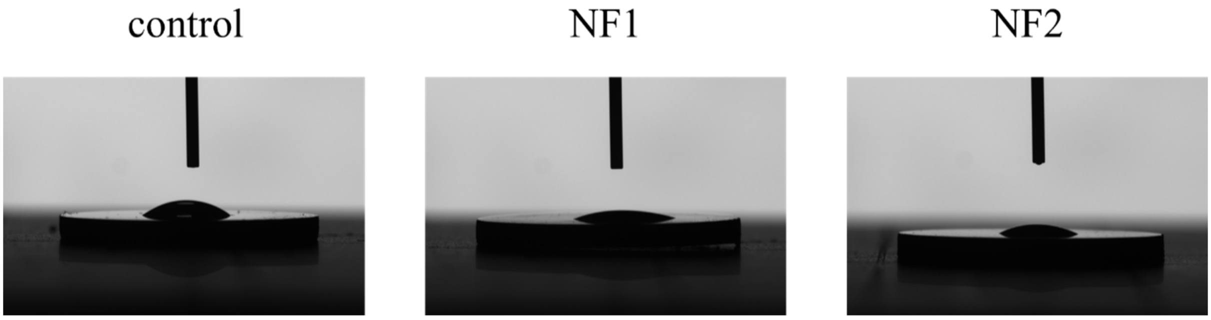

Control	NF1	NF2
46.1±2.6°	16.7±0.7°	26.3±0.8°

Figure S2. Representative images of surface wettability for control, NF1 and NF2 surfaces together with water contact angle (θ W) measurements. Data are presented as mean ± SD; n=3.

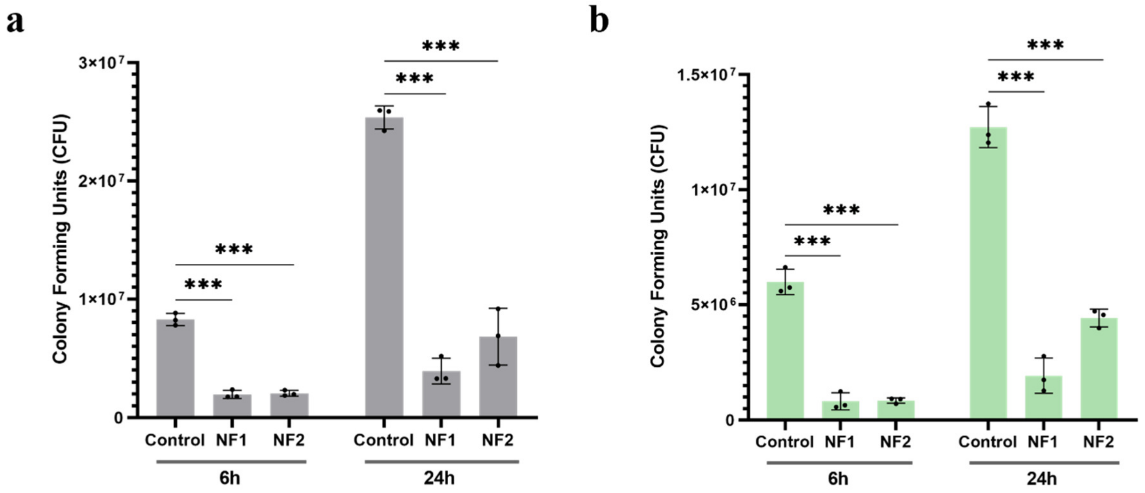

Figure S3. (a) Total number of *E. coli* cells following 6 h or 24 h incubation on nanoflake or control surfaces based on fluorescence intensity. (b) Number of viable *E. coli* cells following 6 h or 24 h incubation on nanoflake or control surfaces. CFU values are given as mean ± standard deviation. *** $P < 0.001$ relative to control, as determined by one-way ANOVA with Tukey HSD post hoc test, $n = 3$. This figure corresponds to Figure 2b in the main text.

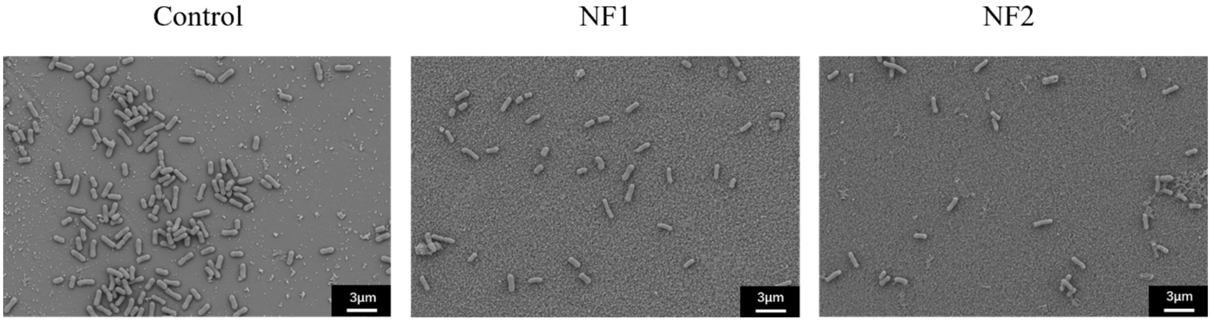

Figure S4. Representative SEM images of *E. coli* on Ti nanoflake surfaces following 6 h incubation.

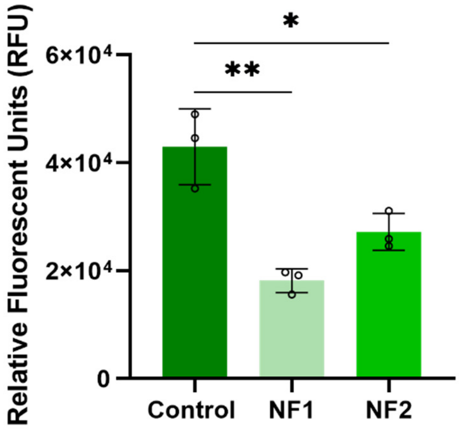

Figure S5. Characterization of *E. coli* biofilms formed on control, NF1 and NF2 surfaces after 24 h incubation. Quantification of total biofilm biomass based on Thioflavin S stain. Data are presented as mean ± SD. * P <0.05 or ** P<0.01 relative to control, as determined by one-way ANOVA with Tukey HSD post hoc test, n = 3.

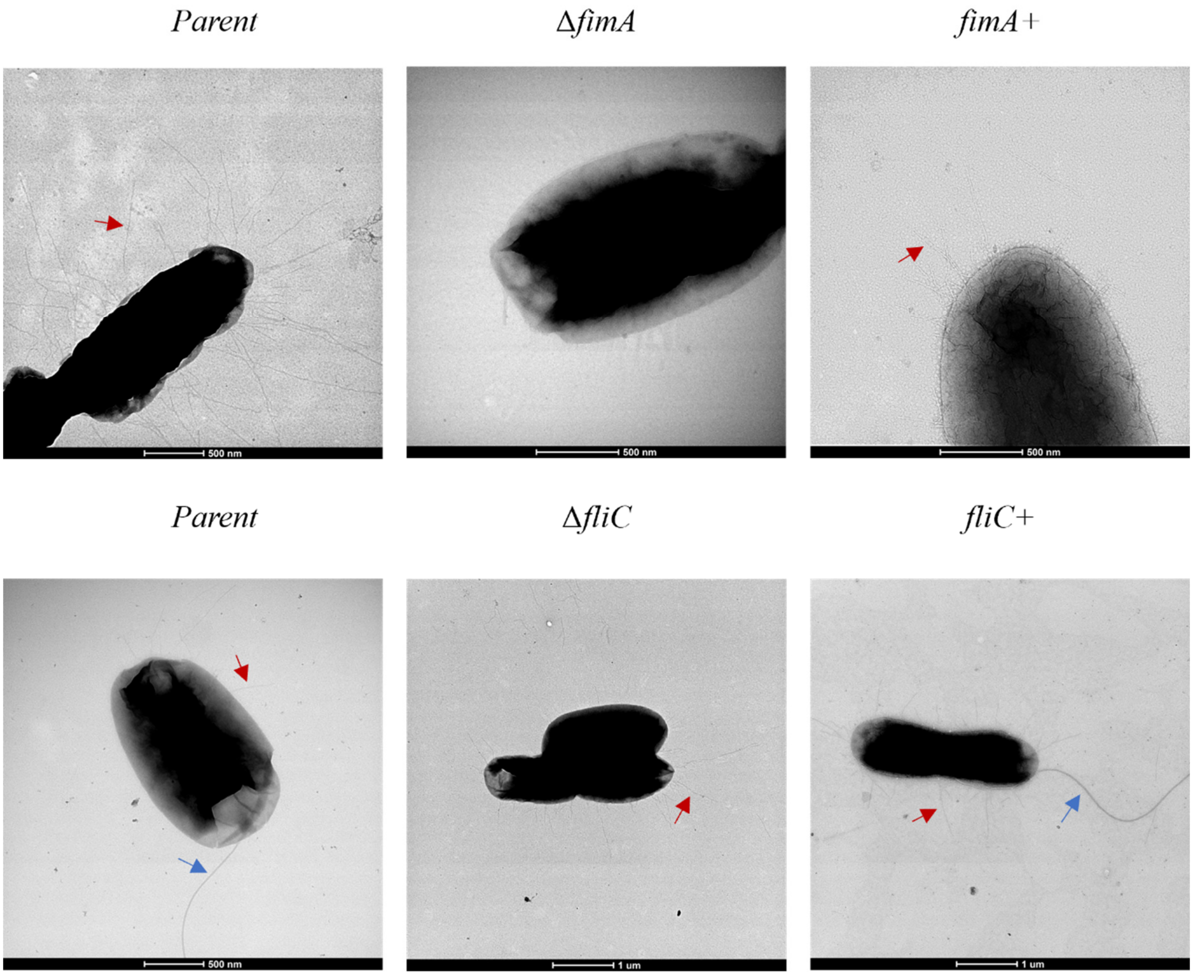

Figure S6. Visualization of parent, Δ*fimA* and *fimA*+ *E. coli* strains (top row) or parent, Δ*fliC* and *fliC*+ *E. coli* strains (bottom row) following negative staining and TEM. Red arrows indicate type-1 fimbriae while blue arrows indicate flagella.

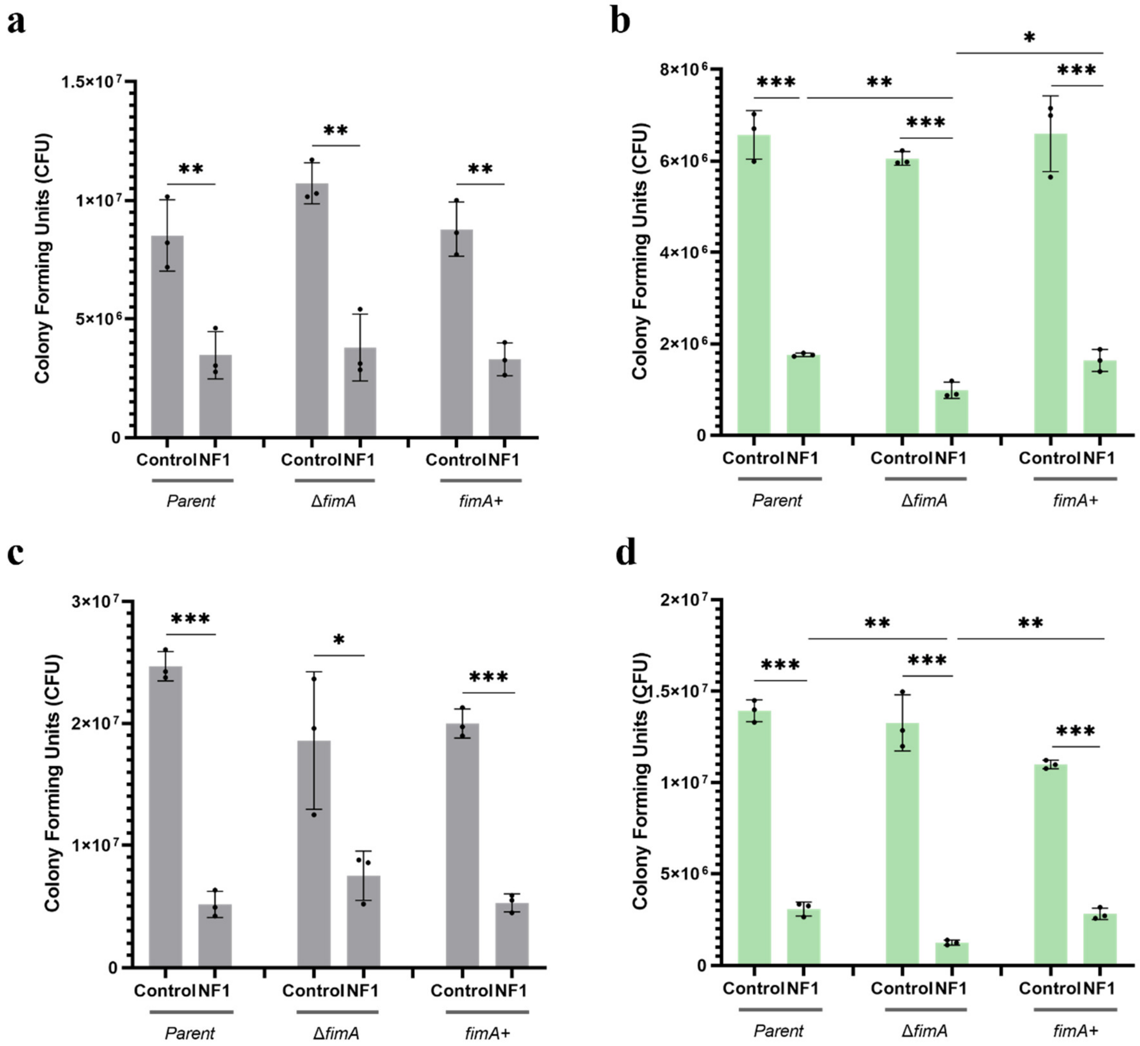

Figure S7. Total number of *E. coli* fimbrial mutant cells following 6 h (a) or 24 h (c) incubation on nanoflake or control surfaces based on fluorescence intensity. (b,d) Numbers of viable bacteria after 6 h (b) or 24 h (d) incubation on nanoflake or control surfaces. CFU values are given as mean ± standard deviation. * $P < 0.05$, ** $P < 0.01$, *** $P < 0.001$ relative to control, as determined by one-way ANOVA with Tukey HSD post hoc test, n = 3. This figure corresponds to Figure 5c and 5d in the main text.

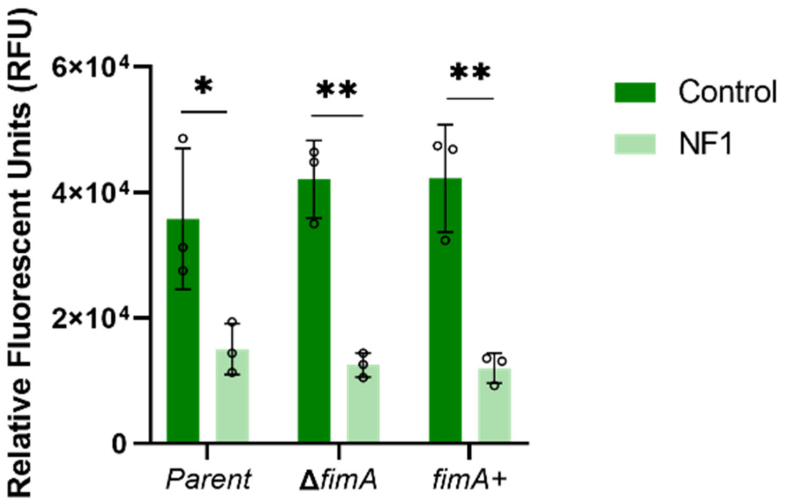

Figure S8. Characterization of *E. coli* parent, Δ*fimA* and Δ*fimA*+ biofilms formed on control or NF1 surfaces after 24 h incubation. Quantification of total biofilm biomass based on Thioflavin S stain. Data are presented as mean ± SD. * $P < 0.05$ or ** $P < 0.01$ relative to control, as determined by one-way ANOVA with Tukey HSD post hoc test, $n = 3$.

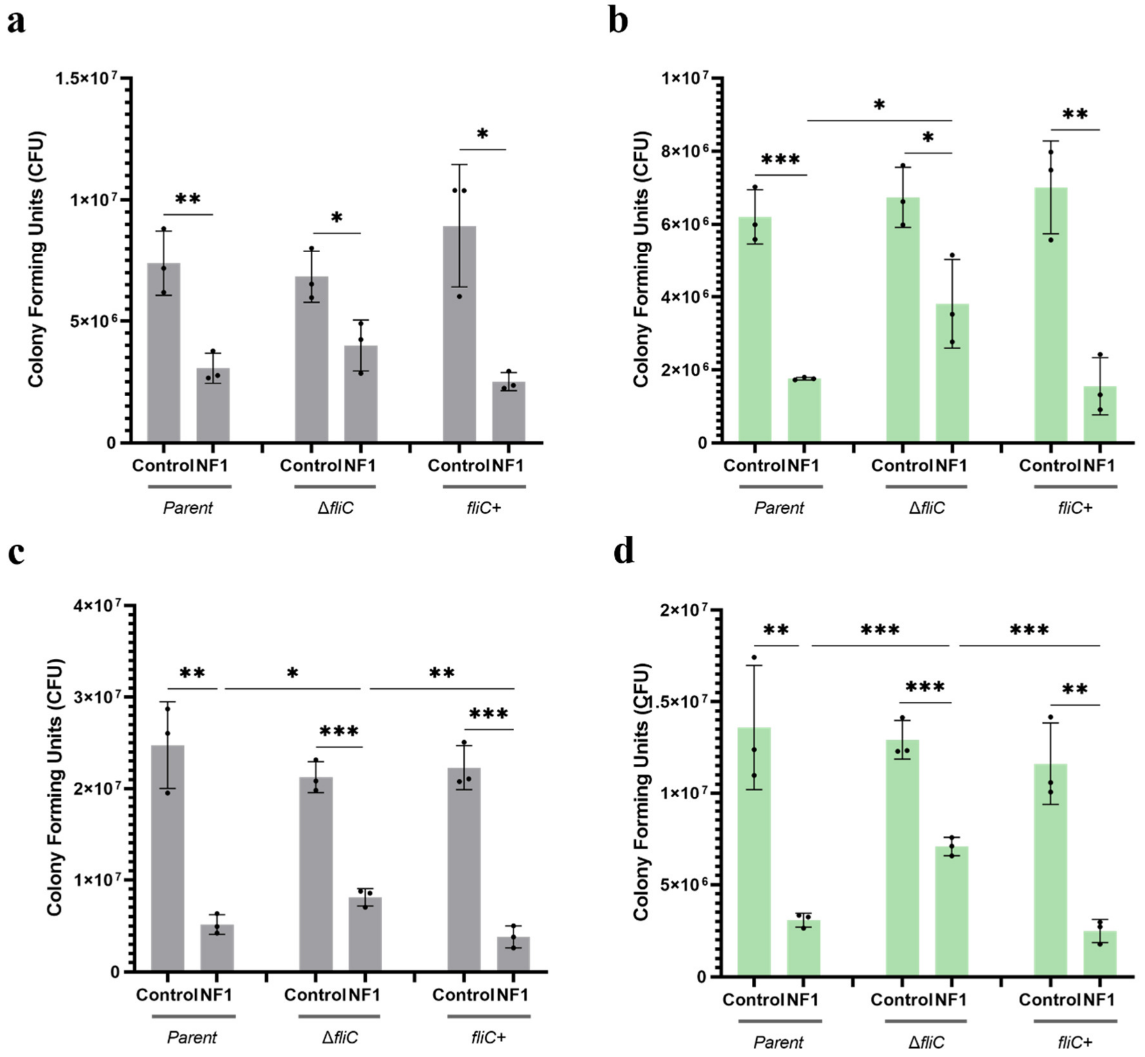

Figure S9. Total *E. coli* flagella mutant cells following 6 h (a) or 24 h (c) incubation on nanoflake or control surfaces based on fluorescence intensity. (b,d) Numbers of viable *E. coli* flagella mutants following 6 h (b) or 24 h (d) incubation on nanoflake or control surfaces. CFU values are given as mean ± standard deviation. * $P < 0.05$, ** $P < 0.01$, *** $P < 0.001$ relative to control, as determined by one-way ANOVA with Tukey HSD post hoc test, n = 3. This figure corresponds to Figure 7c and Figure 7d in the main text.

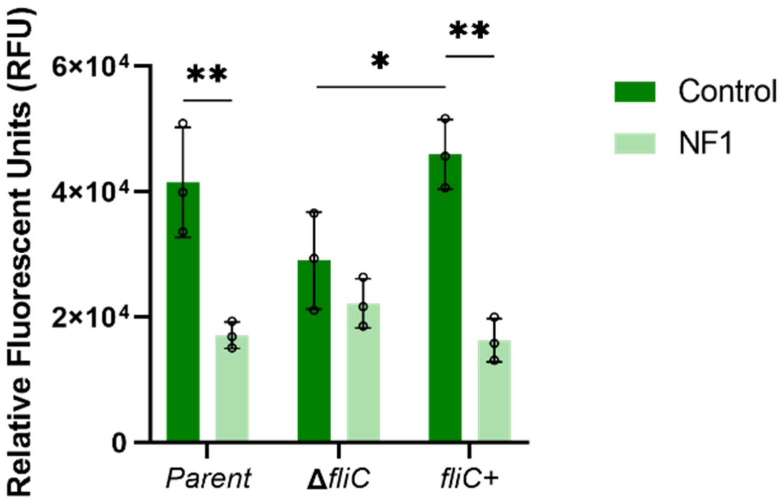

Figure S10. Characterization of *E. coli* parent, Δ*fliC* and Δ*fliC*+ biofilms formed on control or NF1 surfaces after 24 h incubation. Quantification of total biofilm biomass based on Thioflavin S stain. Data are presented as mean ± SD. * P <0.05 or ** P<0.01 relative to control, as determined by one-way ANOVA with Tukey HSD post hoc test, n = 3.

Supplementary References

[1] M. J. Casadaban, *J. Mol. Biol.* **1976,** *104*, 541.

[2] T. Baba, T. Ara, M. Hasegawa, Y. Takai, Y. Okumura, M. Baba, K. A. Datsenko, M. Tomita, B. L. Wanner, H. Mori, *Mol. Syst. Biol.* **2006,** *2*, 2006.0008.

[3] D. J. LeBlanc, L. N. Lee, A. Abu-Al-Jaibat, *Plasmid* **1992,** *28*, 130.

[4] N. M. Thomson, M. J. Pallen, *Curr. Res. Biotechnol.* **2020,** *2*, 45.

[5] P. P. Cherepanov, W. Wackernagel, *Gene* **1995,** *158*, 9.
